# Relationship of Physicians' Rapport with Patients' Satisfaction and Psychological Well-being During Hospitalization

**DOI:** 10.7759/cureus.4991

**Published:** 2019-06-25

**Authors:** Muhammad Mubbashir Sheikh, Rehan Qayyum, Mukta Panda

**Affiliations:** 1 Internal Medicine, University of Tennessee College of Medicine, Chattanooga, USA; 2 Internal Medicine, Virginia Commonwealth University School of Medicine, Richmond, USA; 3 Internal Medicine, University of Tennessee College of Medicine, Health and Science Center, Chattanooga, USA

**Keywords:** patient satisfaction, psychological well-being, hospitalization, physician-patient relationship

## Abstract

Objective

The purpose of this study was to assess the association between the psychological well-being and satisfaction of patients with physicians during their hospitalization.

Methods

This cross-sectional study was conducted at a local hospital using the following surveys: Brief Inventory of Thriving (BIT), tool to assess inpatient satisfaction with care from hospitalists (TAISCH) survey, and Erlanger Internal Patient satisfaction survey addressing demographic questions and questions on physicians’ quality of care and etiquette. Mixed linear regression models were created to examine the effect of psychological well-being on patient satisfaction. Models were adjusted for age, race, and gender, and all analyses were performed in R 3.1.1 using the ‘lme4’ package with statistical significance set at p<0.05.

Results

A total of 360 patients were enrolled in this analysis and the mean age of the cohort was 54.5 years. In the unadjusted analysis, each unit increase in BIT score was associated with a 0.3% (95% CI:0.19-0.4, p<0.001) increase in mean satisfaction score using the five-domain questionnaire and a 0.25% (95%CI:0.16-0.34, p<0.01) increase in mean satisfaction score using the TAISCH questionnaire. Multivariable models, after adjusting confounding variables, also showed the direct and statistically significant relationship between patients’ level of psychological well-being and patient satisfaction. Each unit increase in BIT score was associated with a 0.31% (95% CI:0.20-0.43, p<0.001) and 0.26% (95% CI:0.17-0.36, p<0.001) increase in mean satisfaction scores across the five-domain questionnaire and TAISCH questionnaire.

Conclusions

There is a positive correlation between the level of patients’ psychological well-being and satisfaction with his/her physician with a statistical significance. With patient-specific strategies, we can further improve patient rapport with their physicians, resulting in positive patient outcomes.

## Introduction

Although several financial gains are possible with high patient satisfaction, such as increased reimbursement, decreased malpractice claims, and so on, the most important aspect of patient satisfaction is better patient outcomes [1]. Higher patient satisfaction assists in developing a therapeutic patient-physician alliance, as patients who feel satisfied with their physicians are more likely to follow physician’s recommendations and, in turn, reap health benefits [1]. Patient satisfaction is mainly attributable to the kindness of physicians and nursing staff in comparison to other aspects of hospitalization such as facility accommodations or quality of food [1]. Several studies have examined physician-related factors that determine patient satisfaction and different interventions based on these studies have been suggested to improve patient satisfaction with their hospitalization [2]. However, patient-related factors that determine patient satisfaction have not been examined.

Psychological well-being is a multi-dimensional construct composed of: (1) Hedonic elements (experiencing pleasant events) and Eudaimonic elements (feeling good about self and achieving goals); (2) Emotional aspects (positive and negative emotions, happiness, and life satisfaction); and (3) Purposeful aspects addressing autonomy, environmental mastery, personal growth, positive relationship with others, purpose in life, and self-acceptance [3-4]. The study conducted tested all these elements of psychological well-being in relation to patient satisfaction by utilizing the Brief Inventory of Thriving (BIT) scale tool to assess the inpatient satisfaction with care from hospitalists (TAISCH) questionnaire and the five-domain questionnaire. (Abstract: Muhammad Mubbashir Sheikh, Rehan Qayyum, Mukta Panda. Relationship Between Psychological Well-being and Patient Satisfaction with Physicians During Hospitalization. Hospital Medicine; 2017).

Hospitalization is a stressful event for patients and their families and attending to a patient’s psychological well-being may affect patient outcomes. Illness is associated with lower psychological well-being and, conversely, higher psychological well-being is associated with better health outcomes [5-6]. Furthermore, patients with higher psychological well-being have lower mortality [7-8]. While life events, such as an illness, may adversely affect psychological well-being, certain interventions may increase psychological well-being. Mindfulness, social support, rehabilitation, physical activity, and behavioral therapy are some of the interventions that have been shown to increase psychological well-being in diverse patient populations [9-13]. Patients who experience a higher level of psychosocial well-being are more likely to be optimistic, engaged in healthy behavior, and more contented [14]. Psychological well-being also plays a pivotal role in the development of feelings, ranging from trust to sense of accomplishment, all essential for a healthier, longer, fuller, and happier life [15].

This suggests that patients with high levels of psychological well-being have a higher quality of life and are more satisfied. Also, patients with a higher level of psychological well-being may also tend to transcend their thoughts about their physicians and have more confidence and trust in their physician than patients with psychological distress [5-7]. This makes psychological well-being an influential study in determining the patient-physician relationship.

In addition to patient satisfaction and patient-physician interaction, psychological well-being can also impact patients physically in terms of health outcomes. Patients who exhibit a higher level of psychological well-being benefit from enhanced cardiovascular disease and immunological functions and experience a lower likelihood of suffering from chronic diseases of vessels and immune systems. The higher level of psychological well-being naturally helps in the development of mechanisms that can benefit in the long-term prevention of all-cause mortality and morbidity.

It is possible that patients who have a high level of psychological well-being may have higher satisfaction than those with a low level of psychological well-being. However, this relationship has not been investigated in hospitalized patients. If such a relationship exists between these, then psychological well-being-enhancing interventions can be designed to improve patient satisfaction. Thus, determining if psychological well-being impacts the quality of physician-patient interaction and thus patient satisfaction with physicians may allow a patient-centered approach with an aim to improve the patient's experience with hospitalization. Therefore, the aim of this study was to test an association between a patient’s psychological well-being and patient satisfaction with physicians during hospitalization (Abstract: Muhammad Mubbashir Sheikh, Rehan Qayyum, Mukta Panda. Relationship Between Psychological Well-being and Patient Satisfaction with Physicians During Hospitalization. Hospital Medicine; 2017).

## Materials and methods

This was a cross-sectional study by design, and data points were collected from patients using standardized questionnaires, including the BIT, TAISCH survey, and Erlanger Internal Patient satisfaction survey. The study protocol was approved by the institutional review board at the author’s institution. We included all hospitalized patients at our hospital who were older than 18 years of age, from May 2016 to Sep 2016, and written informed consent for this study was obtained. We excluded individuals who were unable to complete the survey due to illness such as acute delirium or dementia. Nurses and staff were consulted when evaluating whether or not patients were alert, oriented, and able to fill out the survey. Friends and family members were allowed to assist the patients in filling out the surveys but were not allowed to fill out the survey on their own. If a patient was unaware of his or her treating physician, the physician’s name was obtained from the whiteboard in the patient’s room or from the patient’s nurse. Each patient filled out the survey questionnaire only once.

The survey questionnaire consisted of a few demographic questions, a single question asking patients to rate their main treating physician’s quality of care, a validated patient satisfaction tool for hospitalized patients (TAISCH survey), and a validated psychological well-being scale (BIT). The quality rating of treating physician was scored on a five-point (Likert scale; excellent=5 to poor=1). The TAISCH scale consists of 15 items, and questions in this scale include a patient’s perception of a physician's behavior and professional characteristics. The BIT scale consists of 10 items rated on a five-point Likert scale. TAISCH and BIT were scored as recommended by the developers of these scales. Data were summarized using the mean (standard deviation) for continuous variables or frequencies/percentages for categorical variables. To examine an association between psychological well-being and patient satisfaction, we first used linear regression models with and without adjusting for the age, sex, and race of the patient. To take into account the effect of correlation between observations from patients treated by the same physician, we used mixed linear models using physician as the random effect in the unadjusted and adjusted models. All analyses were performed in R 3.1.1 using the ‘lme4’ package and p<0.05 was considered statistically significant. (Abstract: Muhammad Mubbashir Sheikh, Rehan Qayyum, Mukta Panda. Relationship Between Psychological Well-being and Patient Satisfaction with Physicians During Hospitalization. Hospital Medicine; 2017).

## Results

Of the 357 patients, 199 (55%) were females and 47 (13.1%) were African Americans. The mean age of the cohort was 54.5 years (range: 18-93). These patients were seen by 122 physicians and the median number of patients per physician were two (range: 1-13). The raw mean and total BIT scores were 3.854±0.8 and 38.4±18, mean and total TAISCH scores were 3.79±0.79 and 54.9±13, and mean and total five-domain questionnaire scores were 3.9±0.9 and 19.6±4.9, respectively. As expected, there was an inverse correlation between BIT score and age (correlation: -0.10; p=0.06); that is, with increasing age, there was a decrease in the overall psychological well-being but the relationship was not statistically significant. However, there was no difference between African Americans and Caucasians in mean BIT score. Age, sex, and race were not associated with patient satisfaction scores whether measured by the five-domain questionnaire or by the TAISCH questionnaire (p>0.05).

Linear regression

**Figure 1:**
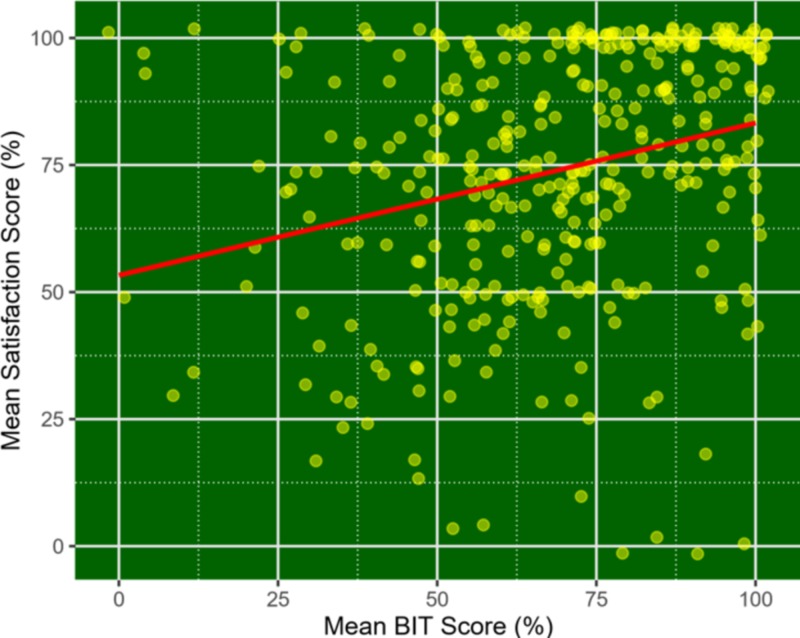
Scatter plot between mean BIT scores and TAISCH questionnaire with superimposed regression lines. Brief inventory of thriving (BIT); tool to assess inpatient satisfaction with care from hospitalists (TAISCH)

**Figure 2:**
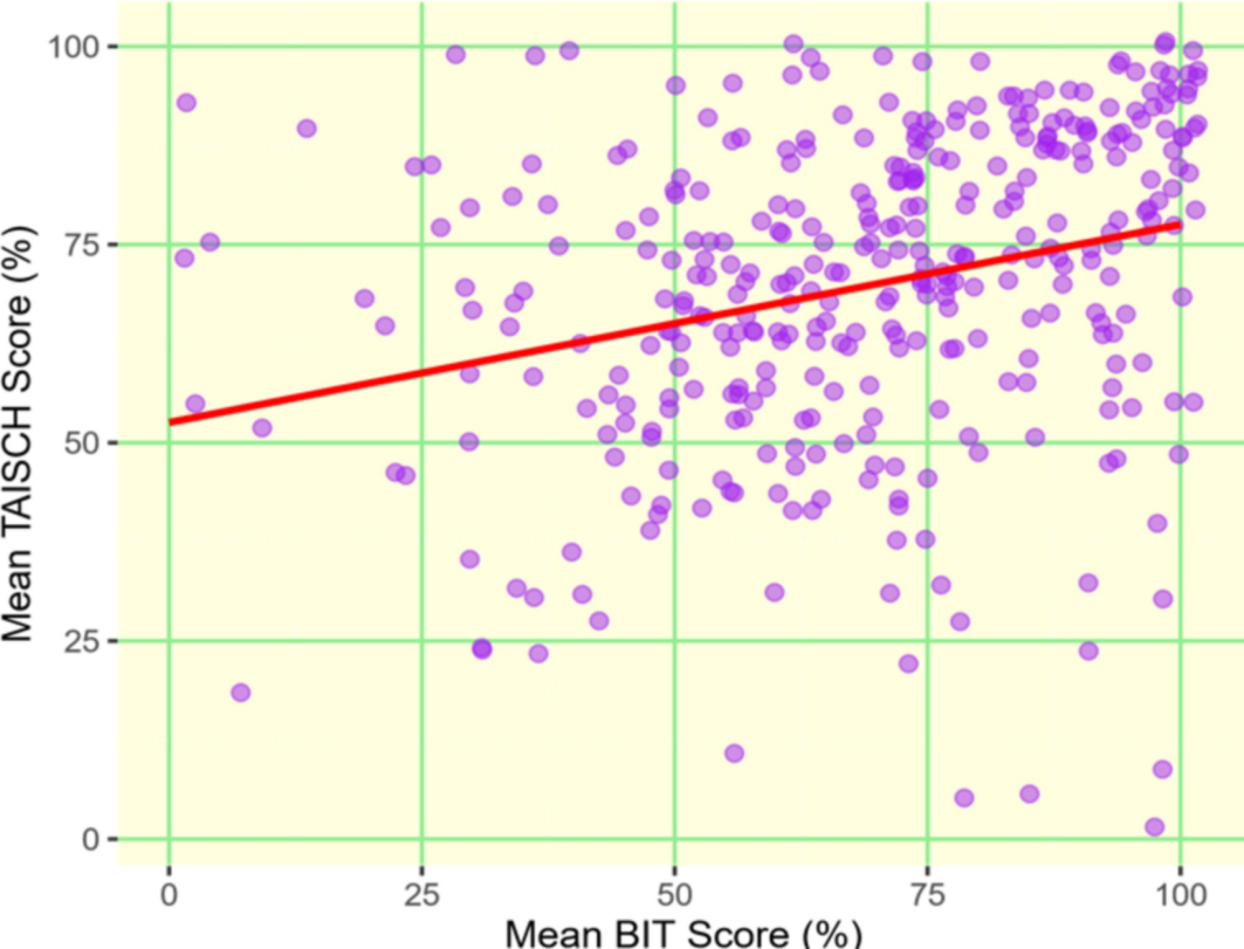
Scatter plot between mean BIT scores and TAISCH questionnaire and five-domain questionnaire Brief inventory of thriving (BIT); tool to assess inpatient satisfaction with care from hospitalists (TAISCH)

In the unadjusted analysis, we found a strong positive association of patient satisfaction with the BIT score (Figures 1-2). After adjusting for the patient’s age, sex, and race, the mean BIT score remained significantly associated with patient satisfaction on both questionnaires (Table 1). Each 1% increase in BIT score was associated with a 0.27% (95% CI: 0.17% - 0.35%; p<0.001) increase in TAISCH score and a 0.31% (95% CI: 0.19% - 0.42%; p<0.001) increase in five-domain questionnaire scores. These results did not change when we replaced mean scores with total scores for the three questionnaires (Table 1).

**Table 1:** Results of linear regression and linear mixed models examining relationship between patient satisfaction and BIT scores. *p-value < 0.001; brief inventory of thriving (BIT), tool to assess inpatient satisfaction with care from hospitalists (TAISCH)

Variables			TAISCH Questionnaire Mean Difference (95% CI)	Five-Domain Questionnaire Mean Difference (95% CI)
Mean Scores	Linear regression	Unadjusted	0.2498	0.2998
		Adjusted	0.265127*	0.31195*
	Linear mixed models	Unadjusted	0.2521	0.301
		Adjusted	0.26491*	0.3134*
Total Scores	Linear regression	Unadjusted	0.2017	0.2651
		Adjusted	0.20872*	0.273475*
	Linear mixed models	Unadjusted	0.2015	0.2632
		Adjusted	0.2056*	0.2732*

Linear mixed models

In the first model, we did not include any covariates and thus the model was consistent with the variance components model, which allowed us to partition variance in patient satisfaction scores between variation that was due to physicians and variation that was not due to physicians. We found that only 0.7% and 6.1% of the variation in patient satisfaction scores on the TAISCH and five-domain questionnaires, respectively, was due to physicians. The results of the unadjusted and adjusted linear mixed models were similar to the linear mixed models and found a direct and statistically significant association between patient satisfaction scores and BIT scores (Table 1). Further, the results did not change when we replaced mean scores with total scores.

## Discussion

Patient satisfaction is not only utilized commonly as a quantification of the quality of medical care delivered but has also become an increasingly consequential component of the delivery of health care. Patient satisfaction depends not only on the quality of care patients received during hospitalization but also relies highly on the patient’s specific characteristics [16]. The aim of this study was to predict or assess the impact of the patients’ level of psychological well-being specifically on the quality of physician-patient interaction and patient satisfaction with physicians during hospitalization. The results analyzed demonstrated a positive and significant correlation between psychological well-being and patient satisfaction with hospitalization on all three scales (p<0.001).

Considering sample characteristics, we explored the possible effects of gender and age. Although males, as compared to females, showed a higher level of psychological well-being as measured by the BIT scale, the results were not statistically significant. This finding relates to the results of previous studies where females reported significantly lower levels of psychological well-being as compared to males [17]. Similar to the previous investigation, we found an inverse relationship between psychological well-being and increasing age, however, the results were not statistically significant [4]. The possible explanation for the decrease in psychological well-being with age can be related to certain co-morbid conditions prevalent at older ages, leading to an increase in the level of depression, in turn, impairing psychological health [18-19].

Psychological wellbeing and health-related quality of life (HRQoL) are closely related. HRQoL is a subset of a broader term known as patient-reported outcomes (PRO) and is a useful method for assessing the patient’s perception of disease symptoms, treatment side effects, and functioning in multiple life domains, such as subjective assessment of physical and social health and psychological well-being. Our findings, along with the results of previous studies evaluating the relationship between psychological well-being and patient-reported outcomes, suggest that psychological factors may influence the patient’s perception of medical care provided to them as reflected by the patient satisfaction scores [20-21].

The results of this study also showed that there is a highly statistically significant positive relationship between the patient’s level of psychological well-being and patient satisfaction with his or her physician. Psychological well-being showed to have between a 26% and a 31% influence on patient satisfaction with his or her physician whereas the physicians themselves only have a 0.7%-6.1% influence. This variation suggests that multiple factors, including many outside the control of physicians, may influence patient satisfaction. Psychological well-being is probably just one of many factors that play a role in determining patient satisfaction. Other previous studies are also in line with our results, which showed that the patients’ perception of good communication with their physician was a more important factor than the physicians’ communication skills [22]. These findings are highly clinically significant as well and demonstrate the need to a push toward patient-centered medicine and personalized patient care. All these factors should be taken into account while using patient satisfaction ratings for administrative decisions.

Clinical significance may be particularly true for psychological well-being where mounting research documents that it can impact patients physically in terms of health outcomes. Patients who exhibit higher levels of psychological well-being benefit from enhanced cardiovascular and immunological functions and experience a lower likelihood of suffering from chronic diseases of the vessels and immune system. The higher level of psychological well-being naturally helps in the development of mechanisms that can benefit in the long-term prevention of all causes of mortality and morbidity [8] and aid in health-promoting behavior such as the implementation of healthy dietary measures, more focus on increasing physical activity, and avoidance of smoking [23-24]. Some published data reported that psychological well-being is inversely associated with illness as a consequence of illness. Patients with low or normal levels of thyroid stimulating hormone (TSH) and deficient in growth hormone (GH) express significant impairment in psychological well-being in terms of social relationships, sexual life, and job satisfaction as compared to normal controls of similar age and sex [5-6]. Another study published shows that promoting and assessing psychological well-being on a wide scale can help in improving the end-of-life care issues and long-term implications of genetic diseases on quality of life [25].

Psychological well-being can also influence treatment success during the disease course of patients. Several studies have been done to highlight the importance of interventions aimed at psychological well-being to improve the health outcomes [13]. When patients with GH deficiency received active treatment for a period, they expressed robust psychological health regarding emotions and energy [26]. The unique study done on cancer survivors shows that compassion-based interventions (CBCT) can improve the psychological well-being and patient-physician relationship and help reduce the mental stress associated with the diagnosis [27]. The use of psychological and psychosocial interventions can improve depressive symptoms in patients diagnosed with a chronic medical illness and are also promising in avoiding future physical health adversity. Mindfulness-based interventions are also increasingly used in both clinical and non-clinical settings to improve both the physiological and psychological aspects of life. Mindful meditation was found to be really helpful in reducing the physical stress in clinical settings and improving the positive emotions of daily life in non-clinical settings [12].

This investigation has several limitations. It is a single center study, which discusses its external validity. Another limitation is the cross-sectional nature of this study, lacking the predictive ability of a longitudinal study. However, the strength of the relationships found here does suggest the potential for the findings to be replicated. To address the issue of confounding patient variables, we used multivariable models. We collected data on relevant variables and adjusted for these variables in our analyses.

## Conclusions

We found a statistically significant direct association between the BIT score and patient satisfaction with physicians during hospitalization. These results identify a significant step towards understanding how to address a patient’s psychological well-being. Mounting research supports the health-protective features of psychological well-being in reducing the risk of disease and promoting the longevity of life. These findings will add to the current research developing different methods of training physicians in meeting psychological well-being. Our results suggest that addressing the psychological well-being of patients during hospitalization may improve satisfaction with their physicians. In light of previous studies, this issue can be addressed by the application of different strategies such as good organization team leadership, the encouragement of health professionals regarding the involvement of patients in making decisions about their health, the employment of psychological and psychosocial education and interventions, improving the physical environment of institutions, and enhancement in resilience training.
